# Large numbers of patients are needed to obtain additional approvals for new cancer drugs: A retrospective cohort study

**DOI:** 10.1038/s41598-023-42213-y

**Published:** 2023-09-26

**Authors:** Charlotte Ouimet, Nora Hutchinson, Catherine Wang, Carol Matyka, Joseph C. Del Paggio, Jonathan Kimmelman

**Affiliations:** 1 Department of Equity, Ethics and Policy, McGill University School of Population and Global Health, 2001 McGill College Avenue, Montreal, QC H3A 1G1 Canada; 2 CARE Advocates Network, Boston, MA USA; 3 Department of Medical Oncology, Thunder Bay Regional Health Sciences Centre and NOSM University, Thunder Bay, ON Canada

**Keywords:** Drug development, Drug development

## Abstract

Patients endure risk and uncertainty when they participate in clinical trials. We previously estimated that 12,217 patient-participants are required to bring a new cancer drug to market. However, many development efforts are aimed at extending the label of already approved drugs. Herein, we estimate the number of patients required to extend the indication of an FDA approved cancer drug. We identified all anti-cancer drugs approved by the FDA 2012 to 2015. We searched clinicaltrials.gov to identify all drug development trajectories (i.e., a series of one or more clinical trials testing a unique drug-indication pairing) launched after FDA approval for each drug. We identified which trajectories produced the following milestones: secondary FDA approvals, secondary FDA approvals achieving substantial clinical benefit in ESMO-MCBS, and recommendations in NCCN clinical practice guidelines. Using the total enrollment, we estimated the number of patients needed to reach each milestone. Forty-two drugs were approved by the FDA between 2012 and 2015, leading to 451 post-approval trajectories enrolling 129,548 patients. Fourteen secondary FDA approvals were identified, of which 4 met the ESMO-MCBS definition of substantial clinical benefit. Fourteen NCCN off-label recommendations were obtained. A total of 9253, 32,387 and 4627 patients were needed to attain an FDA approval, an approval with substantial clinical benefit on ESMO-MCBS, and an NCCN guideline recommendation, respectively. The number of patients needed to obtain a first secondary FDA approval was 16,596. Large numbers of patients are needed to extend the label of prior FDA approved drugs. Label extension after approval entails lower marginal costs for developers. However, extra knowledge available to researchers about a drug’s safety and pharmacology after FDA approval does not appear to translate into reduced patient numbers required for developing new cancer applications.

## Introduction

Pharmaceutical firms bear risks and costs when they develop new drugs^1^. Much of this derives from the high rates of failure and the lengthy timelines required to conduct clinical trials^2,3^.

Patients play a vital role in supporting clinical development, and they too bear some of the risks and expenses associated with clinical trials. Patients participating in trials are required to make frequent clinic visits, undergo invasive procedures (e.g., phlebotomy, repeat biopsies), and endure side effects of treatments which, at the point of testing, are unproven. Some of these research burdens may be offset by the prospect of clinically meaningful benefits, for both themselves and for future patients. Yet, the principle of clinical equipoise establishes that any direct benefits from trial participation are likely to be limited^4^.

One metric of drug development efficiency that illustrates the magnitude of the burden borne by patients in successful drug development is the number of patients required in novel drug development. We previously reported that 12,217 patient-participants are needed to bring a new cancer drug to market^5^. However, almost a third of cancer drug trials involve efforts aim at extending the label of approved drugs. Label extension research is a narrow form of drug repurposing^6^.

Label-extension efforts—especially those pursued after FDA approval (the focus of the present manuscript)—involve less uncertainty concerning the safety of study interventions or the target of the drug^7^. While this might improve efficiencies relative to pre-approval research, other factors might diminish them. In pre-approval stages, drug companies have very strong incentives to minimize the time and costs of testing a new drug. These goals could be achieved by testing drugs in populations that are most likely to show large responses, including biomarker-selected populations. Diminished incentives to maximize efficiencies in the post-approval setting would be expected to erode gains in efficiency due to diminished uncertainty.

A better understanding of which aspects of drug development are most efficient, from a patient standpoint, can help policymakers, academic medical centres, and researchers balance their research portfolios. It can also be used to help patients and patient advocates maximize the value of their research participation.

In what follows, we report the results of a retrospective cohort study estimating the number of patients needed to extend the label of an approved FDA cancer drug. Secondarily, we examine the number of patient-participants needed to achieve an off-label recommendation in the National Comprehensive Cancer Network clinical practice guidelines (NCCN CPG) and those secondary approvals that are deemed to be of substantial clinical benefit defined by the European Society for Medical Oncology-Magnitude of Clinical Benefit Scale (ESMO-MCBS).

## Methods

### Objectives and definitions

Our primary objective was to estimate the number of patients needed to obtain a secondary approval after a new cancer drug has already received its first FDA approval based on a post-approval trajectory. We defined “secondary approvals” as indications added to the FDA label after the first approval of a drug. A “trajectory” is a series of one or more clinical trials testing a unique drug-indication pairing. We defined a “post-approval trajectory” as a trajectory that started after the first FDA-approval of the drug (i.e. secondary approvals might result from pre-approval or post-approval trajectories; we focused on the latter). Secondary objectives included estimating the number of patients needed to obtain a more permissive development milestone (a new recommendation in the National Comprehensive Cancer Network (NCCN) clinical practice guidelines), and a more stringent milestone (secondary approval with substantial clinical benefit using the ESMO-MCBS^8^) based on post-approval development.

Our study was pre-registered on Open Science Framework (OSF) (see https://osf.io/upe4h/); all deviations from and adjustments to our protocol are described in the supplementary materials.

### Creation of FDA approved drug cohort

We identified all anti-cancer drugs approved from January 1st, 2012 to December 31st, 2015 by searching Drugs@FDA for new molecular entities (NMEs) receiving a first approval for cancer (see Supplementary eMethods 1 online). Supportive medications used for symptom management in cancer were excluded. The 2015 cut-off was selected to allow for 6 years of follow-up for secondary approvals. A six year follow-up was selected based on previous work in which we found that 78.6% of secondary approval for oncology drugs approved between 2005 and 2017 occurred within 6 years^9^. All drugs were classified as either cytotoxic therapy, targeted therapy, immunotherapy, or other (see Supplementary eMethods 2 online).

### Capture and characterization of post-approval trajectories

We next identified all drug development trajectories launched after FDA approval for each drug in our sample. This involved searching ClinicalTrials.gov using drug name synonyms to capture all clinical trials initiated after the drugs’ initial FDA approval and assembling the trials into trajectories (see Supplementary eMethods 3 online), using the NCCN broad indication categories (see Supplementary eMethods 4 online). All trial records were updated on December 10th, 2021. Trials within trajectories that began before the initial approval of a drug but continued after initial approval were excluded (see Supplementary eFig. 1 online). A trajectory was deemed biomarker-enriched if the patient population was selected based on a biomarker directly related to the mechanism of action of the drug (see Supplementary eMethods 5 online). Trajectories were classified as industry-initiated if the first trial in the trajectory was sponsored by industry. The enrollments of all clinical trials in eligible trajectories were recorded. Only clinical trials and FDA approvals beginning within 6 years of each original approval were considered to ensure that every drug in our sample had an equivalent amount of time to reach a milestone.

### Milestone attainment

We assessed the number of trajectories attaining each of the three development milestones. For our primary endpoint, we determined whether trajectories led to an FDA approval by identifying all secondary approvals that occurred within 6 years of initial FDA approval (accelerated or full) for all the drugs in our cohort and searching backward to see if the indication-drug pairings matched any of the post-approval trajectories. From this, we obtained the number of secondary approvals stemming from post-approval trajectories.

Second, we determined whether trajectories led to an FDA approval with substantial clinical benefit, as measured by the European Society for Medical Oncology-Magnitude of Clinical Benefit Scale (ESMO-MCBS)^8^. Many FDA approvals are of uncertain or limited clinical impact^10^. To assess the benefit of each FDA approval, we identified the pivotal trial cited in Section 14 of the FDA label of each secondary approval and found the published ESMO-MCBS scorecards^11^. An oncologist (JDP) performed the grading evaluation for all trials without an available scorecard. For secondary indications that were approved via the accelerated approval pathway, if an updated pivotal trial was available up to 5 years after the initial approval of the given indication, this trial was used as part of the ESMO-MCBS grading evaluation.

Third, using the method described above, we determined whether trajectories led to a recommendation in NCCN CPG by searching all NCCN CPG from 2012 to 2021 for instances in which an off-label recommendation for a drug in our sample originated from a post-approval trajectory.

In consultation with a leader of a patient advocacy organization (CM), we also assessed the number of patients needed to attain three additional patient-centred research milestones: a secondary FDA approval supported by a pivotal trial that (a) used randomization, (b) used a clinical endpoint (e.g.: overall survival), (c) measured quality of life. For indications that were approved via the accelerated approval pathway, we evaluated the same trials which were identified for the ESMO-MCBS assessment. For drugs with off-label NCCN recommendations resulting from a post-approval trajectory, the cited supporting evidence was identified and examined for the above properties.

### Analysis

Our primary outcome was calculated by dividing the number of patients enrolled in all post-approval trajectories by the number of secondary FDA approvals. The same calculation was conducted for our secondary outcome milestones. We also evaluated the number of patients needed per approval for industry-initiated vs non-industry initiated trajectories. We performed a descriptive analysis of all the outcomes performed in this study. Due to the limited number of drugs obtaining secondary approvals, we did not calculate confidence intervals. A 15% sample of clinical trials was double coded for inclusion/exclusion, biomarker enrichment and trajectory assignment, resulting in a cohen kappa of 0.81. This agreement was deemed acceptable, and the remainder of the sample was single-coded as per the protocol. All drug types were double-coded by CO and CW and disagreements were resolved through discussion. FDA approval information and off-label NCCN recommendations were singly extracted.

A planned sensitivity analysis that restricted our sample to drugs with longer post-approval follow-up time since FDA approval (8 years) could not be completed because none of the drugs achieved any secondary approvals beyond 6 years of initial approval. The maximum amount of time between initial approval and secondary approval was 5.8 years.

### Post-hoc analyses

We performed a post-hoc analysis evaluating the proportion of approved indications for rare diseases. An indication is “rare” if it has an incidence of less than 6 per 100,000^12^. Cancer.net was used to find the projected number of cases for 2021 for each indication. When unavailable, we obtained estimates from recent publications. Incidence was calculated using the population of the United States on December 10th, 2021 (332 328 876)^13^.

To enable a direct comparison with prior work evaluating the number of patients needed to obtain a first FDA approval^5^, we also determined the number of patients needed to obtain a first secondary approval. Note that this is slightly different from the primary endpoint in the present paper, which included all secondary approvals (not merely the first one).

## Results

We captured 42 cancer drugs that received their first FDA approval between January 2012 and December 2015. Over three quarters were targeted agents (36/42, 86%); 3 were immunotherapy (7%), and 3 were cytotoxic therapy (7%). Four drugs (9.5%) in our cohort received at least one secondary FDA approval resulting from a post-approval trajectory, amounting to 14 secondary approvals (see Table 1). Of these, four (29%) were deemed to present substantial clinical benefits using ESMO-MCBS. Another 14 drug-indication pairings for 5 different drugs were recommended for off-label use in NCCN CPG (see Supplementary eTable 1 online), based on trajectories started within 6 years of approval. The median patient enrollment per drug was 579 patients, with the maximum being 53,547 and the minimum being 6 patients. None of the secondary approvals originated from a biomarker-enriched trajectory. Our findings did not indicate that secondary approvals were restricted to rare diseases; 50% of approvals were indications in this category.

**Table 1:** Secondary FDA approvals resulting from post-approval development.

Drug	Drug class	Initial approval	Secondary approval	Patient enrollment in trajectory	ESMO-MCBS score^a^
Trifluridine and tipiracil	Cytotoxic	Colorectal cancer	Gastroesophageal junction adenocarcinoma	259	3
			Gastric cancer	259	3
Nivolumab	Immunotherapy	Melanoma	Urothelial carcinoma	1139	A
			Malignant pleural mesothelioma	1463	3
Pembrolizumab	Immunotherapy	Melanoma	Head and neck squamous cell cancer	1691	4
			Hodgkin lymphoma	545	4
			Primary mediastinal large B-cell lymphoma	481	3
			PDL1 (CPS ≥ 1) cervical cancer	309	4
			Hepatocellular carcinoma	1632	1
			Merkel cell carcinoma	650	3
			Cutaneous squamous cell carcinoma	797	3
			Endometrial carcinoma	1875	3
			Tumor mutational burden-high (TMB-H) solid tumor	2104	3
Ibrutinib	Targeted therapy	Mantle cell lymphoma	Graft vs. host disease	238	N/A

^a^See Supplementary eTable 3 for PMID of the publications used for all ESMO-MCBS evaluations. An approval is deemed to present substantial benefit with a score of 4–5 or A-B.

**Figure 1:**
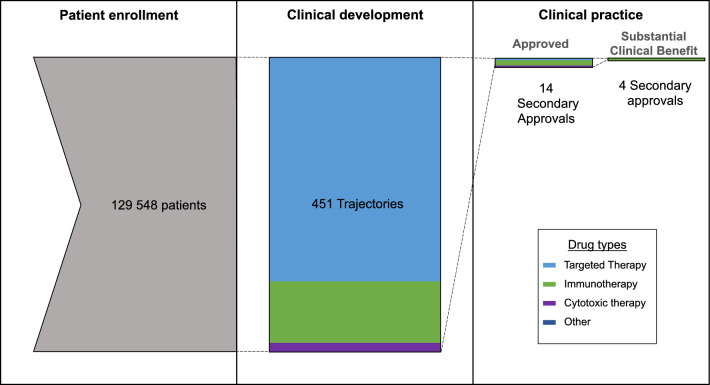
Patient contribution and clinical success of label extension efforts post-approval. The left panel represents the total patient enrollment in all eligible post-approval trajectories. The middle panel represents the trajectories all these patients participated in and the distribution of trajectories by drug type. The right panel represents the approvals that resulted from patient involvement in post-approval development.

A total of 451 post-approval trajectories were recorded for all drugs in our cohort. As indicated in Table 2, 24% of trajectories were biomarker enriched. Of the 451 trajectories captured in our study, 14 (3.1%) resulted in a secondary approval (see Fig. 1), none resulting from biomarker-enriched trajectories. The median patient enrollment per trajectory was 47 patients.

**Table 2:** Properties of post-approval trajectories.

		Number of trajectories
Trajectory property	Industry initiated	66 (18%)^a^
	Biomarker enriched	107 (24%)
Trajectory outcomes	Secondary FDA approval	14 (3%)
	NCCN off-label recommendation	14 (3%)
	Secondary FDA approval presenting substantial clinical benefit (ESMO-MCBS)	4 (1%)
Trajectory drug type	Immunotherapy	97 (22%)
	Cytotoxic therapy	15 (3%)
	Targeted therapy	339 (75%)

^a^Clinical trials for mixed malignancies were omitted from this calculation.

### Patient-enrollment needed to obtain milestones

A total of 129,548 patients participated in post-approval trajectories for drugs in our sample. For our primary outcome, 9253 patients were needed in post-approval clinical trials to obtain a secondary FDA approval. When restricting analysis to only those secondary approvals deemed to have a substantial benefit by ESMO-MCBS, the figure was 32,387. We found that 4627 patients were needed to obtain either an NCCN off-label recommendation or a secondary FDA approval. A total of 16,596 patients were needed for a drug to obtain its first secondary approval.

Of all patients who enrolled in post-approval drug development trajectory trials, 13,440 (10%) were enrolled in trials within trajectories that advanced to one of the 14 FDA approval and 3683 (2.8%) in trajectories leading to secondary approvals with substantial clinical benefit. When only considering the patients enrolled in trajectories prior to the first secondary FDA approval for all the drugs in our cohort that experienced label extension, 5.4% of patients directly contributed to the 4 first secondary approvals.

### Patient enrollment by research strata and evidence quality milestones

Of the 14 trajectories that resulted in a secondary approval, seven (50%) were industry initiated. A total of 8954 patients were needed per approval for industry-initiated trajectories vs. 9553 patients for non-industry-initiated trajectories.

By December 10th, 2021, 5 (63%) of 8 accelerated approvals reported the results of a confirmatory pivotal trial on the FDA label within 5 years of approval (see Supplementary eTable 2 online). A total of 16,193 patients were needed to obtain a secondary approval that was based on a randomized clinical trial, 21,591 patients were needed to obtain an approval based on a trial that used a survival endpoint, and 32,387 patients were needed to obtain a secondary approval that was based on a clinical trial that measured health-related quality of life. None of the NCCN off-label recommendations that occurred within 6 years of initial approval were based on trials that used randomization, a clinical endpoint, or quality of life measures (see Supplementary eTable 2 online).

## Discussion

In this retrospective cohort study, we found that large numbers of patients are needed to extend the label of already approved cancer drugs. In particular, 9253 patient participants are needed to obtain a new label for a prior FDA-approved drug, and 32,387 patient participants are needed to obtain a secondary approval deemed to be of substantial clinical benefit by ESMO-MCBS. The number of patients needed to obtain either an NCCN recommendation for off-label use or a secondary FDA approval was considerably lower: 4627. Our findings do not suggest a difference in patient volume needed for a secondary approval for research efforts initiated by industry compared to non-industry-initiated efforts. They do suggest, however, that industry-initiated trajectories are more fruitful: although 18% of trajectories are industry-initiated, 50% of approvals result from such trajectories.

Efficiencies associated with pre-approval trajectories were evaluated in a separate study using a slightly different time period, but we think the methods and time period are similar enough to support cautious statements about efficiencies in drug development pre vs. post-approval. Our present analysis suggests that the number of patients needed to obtain a first secondary approval based on post-approval development (16,596 patients) is similar to the number of patients needed to obtain a first FDA licensure (12,217 patients)^5^. On a per-patient basis, 5.4% of patients in our sample directly contributed to the trajectories of a first secondary approval, as compared with 19% for the first approval of a new drug^5^. That post-approval development is likely to be no more efficient, on a per-patient basis, than in initial indication/drug discovery suggests that whatever efficiencies gained from greater knowledge of mechanism and safety are offset by lower prior probabilities of achieving regulatory approval when initiating testing of new clinical hypotheses after a drug is already approved. Or, they could be offset by drug companies having stronger incentives to minimize patient enrollment in pre-license trials through the use of accepted biomarkers. However, this interpretation is somewhat inconsistent with our finding that the proportion of trajectories employing biomarker enrichment was 24%, which is the same proportion reported for pre-license trials^5^. Our findings align with prior studies of post-approval development. In a study of cancer drugs approved 2005–2007, no new FDA approvals were obtained from 69 disease-indication pairings that were launched into trials^14^. Another analysis found that of 60 secondary approvals occurring within 6 years of initial licensure for cancer drugs approved from 2005 to 2017, 9 (15%) resulted from post-approval development^9^. Another report suggested that label extensions are less medically impactful than initial drug approvals, as measured by effect sizes or disease prevalence of approved indications^15^. Our findings cannot answer the question of whether this reflects diminished prior probability on scientific hypotheses tested after approval, or diminished commercial investment in post-approval trials and regulatory submission.

The metric of drug development efficiency used in the present manuscript is intended to better track the most morally significant features of drug development^5,16,17^. Human protection policies urge that risks be minimized. Moreover, patients in trials are likely to have strong preferences for participating in trials that have the greatest prospect for improving options for future patients. Metrics of efficiency conventionally used in drug development, like the number of failed compounds per FDA approval^2^, phase transition rates^18^, or amount of financial investment per FDA approval^19^, capture aspects of the drug development that are more likely to matter for pharmaceutical sponsors rather than for patients. By focusing on per patient impact on FDA approvals, we do not intend to suggest that label extension efforts are necessarily inefficient using more sponsor-centered metrics. Nor do we rule out that efficiencies using sponsor-centered metrics have indirect benefits for patients, such as lowered drug costs or, in some cases, earlier treatment access.

Our study should be interpreted in light of several limitations. First, our measure of the relationship between volunteerism and impact is simple. The number of patients does not account for the intensity of research burdens, and FDA approval is a crude measure of practical impact. However, we did evaluate the clinical benefit of secondary approvals using ESMO-MCBS to better understand the impact of the approvals we captured. Second, our cohort of cancer drugs is limited to those approved by the FDA from 2012 to 2015 and may not reflect the patient burden of recently approved cancer drugs. Third, we only consider secondary approvals and NCCN off-label recommendations that occur within 6 years of approval. Fourth, we could not perform our intended subgroup analysis stratifying our primary endpoint by biomarker enrichment versus non-biomarker enriched. Were we able to perform this analysis, we would have expected to observe that biomarker-enriched trajectories are more efficient, as was observed in the study by Hutchinson et al.^5^. We were also unable to perform a planned analysis at 8 years as none of the drugs with 8 years of follow-up achieved label extensions based on post-approval development. Greater follow-up time would be expected to increase both the number of patients captured as well as the number of secondary approvals.

In summary, for cancer drugs receiving approval between 2012 and 2015, 129,548 patients participated in clinical trials initiated after approval to support drug label extension. Our findings reinforce that large numbers of patients enrolled in clinical trials are needed to achieve advancements in cancer clinical research after a drug is already approved. Post-approval trials may be more likely to advance treatment options for rare cancers, and perhaps result in more immediate changes in care for patients. However, findings reported here, juxtaposed with those reported elsewhere, suggest that clinical development efforts pursued after drug approval are no more successful than pre-approval drug development efforts, despite relatively mature knowledge of a drug’s safety and pharmacology.

### Supplementary Information

Supplementary Information.

## Data Availability

The datasets generated and analyzed during the current study are not publicly available due to privacy purposes but are available from the corresponding author upon reasonable request.
