# Supplementary material for: Large numbers of patients are needed to obtain additional approvals for new cancer drugs: A retrospective cohort study
Source: Sci Rep. 2023 Sep 26;13:16138. doi: 10.1038/s41598-023-42213-y (PMC10522579; doi:10.1038/s41598-023-42213-y)
Supplement: Supplementary file 1 — Supplementary Information. [file 41598_2023_42213_MOESM1_ESM.docx]

**Supplementary Methods**

**eMethods1 - Search Details**

We collected drug name synonyms using NCI thesaurus. If no entry was found in the NCI thesaurus, the search was re-performed in PubChem. If synonyms were found in FDA approval documents/clinicaltrials.gov entries, they were added to the list of synonyms. For each drug, the date of the original approval and all subsequent approvals was recorded. Withdrawn approvals were not included in our analysis.

**eMethods2 - Screening Drugs**

We examined the drug description provided on NCI thesauruses and used this information to classify each drug as either cytotoxic, immunotherapy, targeted or other. A drug that predominately stimulates or manipulates the immune system to recognize and/or target cancer cells, was classified as immunotherapy. Targeted drugs inhibit or activate specific molecular targets. Cytotoxic drugs affect all dividing cells and lead to cell death. The model we use is hierarchical which means that if a drug has characteristics for more than one drug class, it will be classified based on the more innovative mechanism. The order of drug classifications from most to least innovative is as follows: immunotherapy, targeted therapy, and cytotoxic therapy. Should a drug not fit into any of the three categories described above, then it was labelled as “other”.

**eMethods3 - Screening Trials**

We contended with instances in which a particular clinical trial tested either multiple drugs or multiple indications as follows: when a trial included 2 different indications, then the trial was treated as reflecting two trajectories (ex: trajectory 1A, trajectory 1B) and patients were evenly assigned to both trajectories. This technique was employed for up to 3 different malignancies. Beyond 3 indications, trials were labelled as either “mixed solid malignancies,” “mixed hematological malignancies” or “mixed solid and hematological malignancies.” Should one of two indications tested in a trial originate from pre-approval development, half of the patients were excluded from the analysis.

For master protocols or instances in which the drug of interest was used in fewer than 50% of the arms, only the patients enrolled in the arm(s) of interest were counted. To obtain an estimate for this number, then the trial enrollment was divided by the number of arms in the study and multiplied by the number of eligible arms.

In instances where a combination trial included two drugs from our sample, the trial was included in both trajectories, but each was considered as 50% of a trial and the number of patients was assigned 50% to one trajectory and 50% to the another to avoid overcounting patients. The same was true if 3 or more drugs are tested; the number of patients was divided by the number of drugs. Should a drug be in combination with a drug outside the scope of our study, then all patients were counted.

When a trial was labelled as a Phase1/2, then the trial was classified according to the indication tested in the Phase 2 section of the trial.

When a trajectory resulted in a secondary FDA approval, only patients enrolled in clinical trials that began before the date of secondary FDA approval were counted.

**eMethods4 - Defining Cancer Indications**

Because many trajectories start in broad indications and narrow to sub-indications as they progress, we assigned each indication to an indication category using the broad indication categories used by the National Comprehensive Cancer Network (NCCN) guidelines. For example, since the guidelines for treating follicular lymphoma and mantle cell lymphoma are provided in NCCN’s guidelines for treating B-cell lymphoma, both these indications fall into the same indication category and are treated as one broad indication. Thus, a trial testing drug X in follicular lymphoma and a trial testing drug X in mantle lymphoma were part of the same trajectory.

**eMethods5 - Biomarker enrichment**

A trajectory was labelled as “biomarker enriched” if all the trials founding this trajectory were enriched. A trial would fulfill the criteria for enrichment if the mechanism of action of the drug tested was directly related to the biomarker for which the patient population was enriched. In this sense, our definition of enrichment resembled that of “predictive enrichment” proposed by the FDA in which a protein or genetic marker related to the drug’s mechanism of action is used to select the patient population.^1^ In instances where a specific drug-indication pairing was observed in both enriched trials and unenriched trials, then two separate trajectories were made for both avenues of development despite the same indication being tested in both.

**Changes to Protocol**

Some of our pre-specified analyses did not, on reflection, align with our main goals and were therefore excluded from the manuscript. This includes a Kaplan-Meier plot comparing time to secondary FDA approvals.

We had planned to assess the number of patients needed to obtain a secondary approval by drug class but did not perform this analysis due to the low number of secondary approvals for some drug classes. We also did not assess the number of patients needed to obtain a biomarker enriched approval since no biomarker enriched approvals originated from a biomarker enriched trajectory.

We had originally planned to exclude mixed malignancy trials from the calculation that is comparing the number of patients per approval for industry-initiated vs non-industry-initiated trajectories. Upon reconsideration, we classified mixed malignancies studies on a trial level as industry-sponsored or not and proceeded to include these patients in our calculations.

We planned to only double code 10% of the sample, but due to available personnel, we double coded 15%.

We had planned to include withdrawn off-label recommendations in NCCN guidelines in our study. For consistency, we chose not to include such recommendations since we did not consider withdrawn FDA approvals in our project.

**Supplementary Analysis**

eTable1: NCCN Off-label Recommendations Stemmed from Post-Approval Trajectories

| **Drug** | **Drug Type** | **Indication** | **Patient Enrollment** |
| --- | --- | --- | --- |
| Cobimetinib | Targeted | CNS Cancer | 36 |
| Lenvatinib | Targeted | Head and Neck Cancer | 304 |
| Nivolumab | Immunotherapy | Anal Carcinoma | 481 |
|  |  | Chronic Lymphocytic Leukemia | 72 |
|  |  | T-cell Lymphoma | 27 |
| Pembrolizumab | Immunotherapy | Anal Carcinoma | 32 |
|  |  | Chronic Lymphocytic Leukemia | 53 |
|  |  | Malignant Pleural Mesothelioma | 569 |
|  |  | Primary Cutaneous Lymphoma | 58 |
|  |  | Soft Tissue Sarcoma | 327 |
|  |  | T-cell Lymphoma | 72 |
|  |  | Thymomas and Thymic Cancer | 148 |
|  |  | Uveal Melanoma | 412 |
| Ibrutinib | Targeted | CNS Cancer | 352 |

eTable2: Property of Pivotal Trials Cited to Support either Secondary FDA Approvals or NCCN Off-label Recommendations

| **Property** | **Original Pivotal Trial Supporting Secondary Approvals** | **Updated Pivotal Trials Supporting Secondary Approvals** | **Trials supporting off-label recommendations in NCCN CPG.** |
| --- | --- | --- | --- |
| Randomization | 21% | 57% | 0% |
| Survival Endpoint | 21% | 43% | 0% |
| Measure Quality of Life (QoL) | 14% | 29% | 0% |
| Substantial clinical benefit (ESMO) | 0% | 29% | N/A |

eTable 3: Publications used for ESMO-MCBS Score Evaluation

| **Drug** | **Secondary Approval** | **PMID First Pivotal Trial** | **Score** | **PMID Updated Pivotal Trial** | **Score** |
| --- | --- | --- | --- | --- | --- |
| Trifluridine and Tipiracil | Gastroesophageal Junction Adenocarcinoma | 30355453 | 3* | N/A | N/A |
|  | Gastric Cancer | 30355453 | 3* | N/A | N/A |
| Nivolumab | Urothelial Carcinoma | 28131785 | 2 | 34077643 | A |
|  | Malignant Pleural Mesothelioma | 33485464 | 3 | N/A | N/A |
| Pembrolizumab | Head and Neck Squamous Cell Cancer | 27646946 | 1 | 31679945 | 4* |
|  | Hodgkin Lymphoma | 28441111 | 3 | 33721562 | 4 |
|  | Primary Mediastinal Large B-Cell Lymphoma | 31609651 | 3 | Publication Pending | - |
|  | PDL1 (CPS ≥1) Cervical Cancer | 30943124 | 3 | 34534429 | 4 |
|  | Hepatocellular Carcinoma | 29875066 | 1 | Publication Pending | - |
|  | Merkel Cell Carcinoma | 33879601 | 3 | Publication Pending | - |
|  | Cutaneous Squamous Cell Carcinoma | 32673170 | 3 | N/A | N/A |
|  | Endometrial Carcinoma | 32167863 | 3 | Publication Pending | - |
|  | Tumor Mutational Burden-High (TMB-H) Solid Tumor | 32919526 | 3 | N/A | N/A |

*Published scorecard

**eFigure1 – PRISMA Flow Diagram for Identification of Cohort of Clinical Trials**

## Identification

## Screening

## Eligibility

## Included

Records identified through database searching^a^
(n = 6223 Trials)

Additional records identified through other sources
(n = 0)

Records excluded^b^
(n = 1177 Trials)

Full-text registration documents assessed for eligibility

(n = 5046 Trials)

Eligible trials

(n = 1688 Trials)

Records screened

(n = 6223 Trials)

- Excluded trials testing initially approved drug-indication pairing (n=1732)
- Excluded trials of drug-indication pairings that were initiated pre-approval (n=1515)
- Excluded trials not testing the drug of interest (n = 43)
- Excluded ex vivo studies (n=1)
- Excluded extension studies (n = 34)
- Excluded trials treating symptoms of cancer (n=11)
- Excluded trials that included healthy participants (n=22)

a) Search of clinicaltrials.gov for clinical trials testing drug from our cohort that were initiated after the date of initial FDA approval of each respective drug

b) We excluded trials that were initiated 6 or more years after the initial approval of each respective drug

**References**

1 Enrichment Strategies for Clinical Trials to Support Determination of Effectiveness of Human Drugs and Biological Products: Guidance for Industry. U.S. Department of Health and Human Services Food and Drug Administration. 2019. (<https://www.fda.gov/media/121320/download>). Accessed 12/12/2019.
